# Abundances of Iron-Binding Photosynthetic and Nitrogen-Fixing Proteins of *Trichodesmium* Both in Culture and *In Situ* from the North Atlantic

**DOI:** 10.1371/journal.pone.0035571

**Published:** 2012-05-01

**Authors:** Sophie Richier, Anna I. Macey, Nicola J. Pratt, David J. Honey, C. Mark Moore, Thomas S. Bibby

**Affiliations:** Ocean and Earth Science, National Oceanography Centre, Southampton, University of Southampton, European Way, Southampton, United Kingdom; Royal Netherlands Institute of Sea Research (NIOZ), The Netherlands

## Abstract

Marine cyanobacteria of the genus *Trichodesmium* occur throughout the oligotrophic tropical and subtropical oceans, where they can dominate the diazotrophic community in regions with high inputs of the trace metal iron (Fe). Iron is necessary for the functionality of enzymes involved in the processes of both photosynthesis and nitrogen fixation. We combined laboratory and field-based quantifications of the absolute concentrations of key enzymes involved in both photosynthesis and nitrogen fixation to determine how *Trichodesmium* allocates resources to these processes. We determined that protein level responses of *Trichodesmium* to iron-starvation involve down-regulation of the nitrogen fixation apparatus. In contrast, the photosynthetic apparatus is largely maintained, although re-arrangements do occur, including accumulation of the iron-stress-induced chlorophyll-binding protein IsiA. Data from natural populations of *Trichodesmium* spp. collected in the North Atlantic demonstrated a protein profile similar to iron-starved *Trichodesmium* in culture, suggestive of acclimation towards a minimal iron requirement even within an oceanic region receiving a high iron-flux. Estimates of cellular metabolic iron requirements are consistent with the availability of this trace metal playing a major role in restricting the biomass and activity of *Trichodesmium* throughout much of the subtropical ocean.

## Introduction

Open-ocean diazotrophic cyanobacteria, such as *Trichodesmium* spp., are of particular importance due to their contributions to the carbon (C) cycle through primary production and to the nitrogen (N) cycle through the fixation of atmospheric N_2_ [1]–[3]. *Trichodesmium* spp. are thought to comprise the most abundant diazotrophic cyanobacteria in the oceans [3], often forming widespread blooms over subtropical and tropical regions and potentially contributing a larger fraction of total marine nitrogen fixation than any other organism [3]–[6]. While marine phytoplankton are N-limited through much of the tropical and subtropical oceans [7]–[9], diazotrophic growth is likely limited by the availability of other nutrients such as phosphorous (P) and iron (Fe) [10]–[15].

Within the photosynthetic apparatus, the chlorophyll-binding membrane protein complexes photosystem II (PSII) and photosystem I (PSI) both have an absolute requirement for iron. Photosystem I (PSI) represents the largest photosynthetic requirement for iron, each PSI trimer containing 36 Fe atoms in 9 [4Fe-4S] clusters [16]. Compared with other autotrophs, photosynthetic diazotrophs, including *Trichodesmium*, have a significant further iron requirement due to the abundance of iron-containing enzymes in the nitrogen-fixation apparatus [17]–[19]. The nitrogenase complex (the key enzyme in N_2_ fixation) is composed of two Fe-proteins encoded by the *nifH* gene, each containing 4 Fe atoms [20] and a dimeric MoFe protein encoded by *nifD* and *nifK* genes, which contains a total of 30 Fe atoms [20], [21], making it one of the most iron-rich enzymes in nature [17], [22]. As a consequence, the availability of iron in marine systems appears to greatly influence N_2_ fixation in cyanobacteria [10], [23]–[26].

Models predict that the distribution of nitrogen fixation in the modern ocean may be constrained by the availability of iron [27]–[29]. Moreover, oceanographic distributions are consistent with the availability of iron affecting N_2_ fixation and, consequently, the biogeography of diazotrophic organisms including *Trichodesmium* [14], [15]. The North Atlantic Ocean has some of the highest N_2_ fixation rates in the global ocean [15], with waters characterized by high dissolved Fe concentrations tightly linked to high atmospheric dust inputs [14], [30]. However, evidence for enhanced N fixation rates following Fe addition to North Atlantic waters suggest that enhanced Fe may influence *Trichodesmium* growth even in this ocean basin [12], [31].

Diazotrophy is a significant challenge for oxygenic photoautotrophic microorganisms because O_2_ is inhibitory to the N_2_ reduction enzyme nitrogenase [32], [33]. Diazotrophs have developed specific molecular and physiological strategies to protect nitrogenase from the O_2_ evolved during photosynthesis [28], [34], [35]. Some diazotrophs have adapted to fix nitrogen during the dark period to avoid photosynthetic oxygen inhibition of the nitrogenase complex (temporal separation) [36]–[39], while others have terminally differentiated cells, termed heterocysts, with thickened cell walls and reduced photosynthetic activity (spatial separation) [40]. The non-heterocystous *Trichodesmium* uniquely undertakes both CO_2_ and N_2_ fixation during the day in the same cell through a complex combination of apparently reversible temporal and intracellular-spatial separation of these processes [4], [34], [35], [41]. The simultaneous occurrence of two iron-rich metabolic processes in *Trichodesmium* may also suggest a higher specific iron requirement per cell than for other diazotrophic organisms [28].

While the impacts of iron on growth, C and N_2_ fixation, O_2_ production and dark respiration in *Trichodesmium* in culture have been well documented [24], [25], [42]–[44], relatively little is known of the molecular adaptation of this organism to iron availability in the environment. Although transcriptomic and protein level responses to iron stress have been observed in culture [19], [45]–[47], information from *in situ* natural populations is limited [48]–[50]. Here we report the responses of photosynthetic and nitrogen fixing proteins to iron availability in laboratory cultures of *Trichodesmium* IMS101 and compare these with natural populations of *Trichodesmium* from the subtropical North Atlantic. We quantified peptides indicative of the abundance of the major iron-binding proteins involved in nitrogen fixation and photosynthesis, including the iron protein of nitrogenase (NifH) and the major chlorophyll-binding proteins in photosynthesis, the D1 protein of PSII (PsbA) and a core subunit of PSI (PsaC). In addition we assessed the presence and accumulation of the iron-stress-induced chlorophyll-binding protein IsiA [51], [52]. Through absolute quantification of these enzymes we characterized the molecular acclimation strategy of *Trichodesmium* to iron availability and hence estimate the iron supplies required to sustain natural populations.

## Materials and Methods

### Sample Collection

#### Culture

*Trichodesmium* IMS101 (originally isolated by [53]) was grown as batch-cultures in YBC-II medium [54], made from artificial seawater filtered through 1.0- and 0.2-µm Millipore membrane filters. Culture conditions included a 12/12-h light-dark cycle using cool white lamps at 50 µmol photons m^-2^ s^-1^ and temperature of 25°C. Small volume cultures (50 ml and 200 ml) were grown in polycarbonate flasks (Nalgene) with gentle agitation on an orbital shaker. Cells from a late-log-phase culture (as estimated by chlorophyll concentration measured daily) were used as an inoculum (1/50 dilution) for five parallel cultures grown in YBC-11 supplemented with 20, 40, 120 and 200 nM Fe (cultures grown in YBC-11 medium with no added iron did not grow). Our culture conditions (2µM EDTA) would not have buffered iron concentrations. Consequently biological iron uptake resulted in the development of starvation within the cultures. Samples were harvested after a 7 to 9-day incubation period, when the 20 nM Fe culture first showed evidence of physiological iron-stress as indicted by reduced photosynthetic efficiencies. Measurements of F_v_/F_m_ were made 2 h after the start of the light period. All nutrient stress experiments were performed as biological triplicates.

#### Field

**Figure 1:**
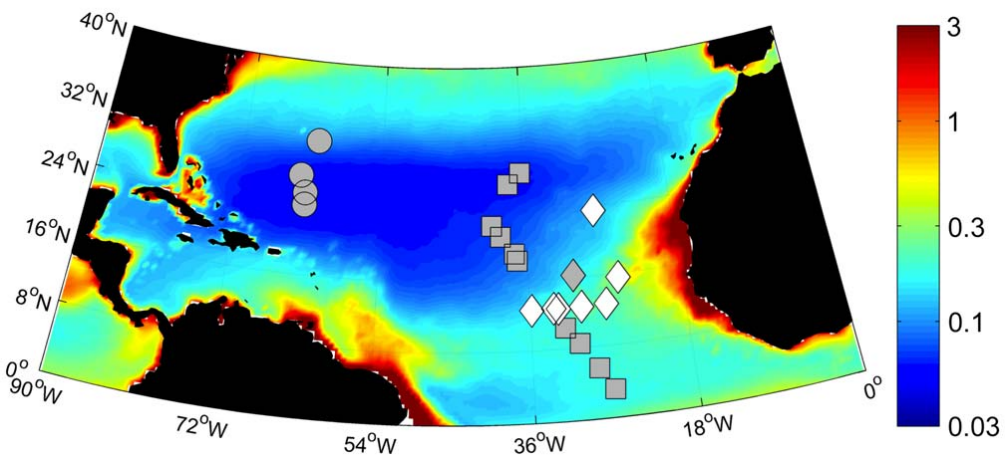
Locations of sampling of *Trichodesmium* colonies in the North Atlantic. Samples were collected during three different cruises: AMT 19 (squares), D326 (diamond) and FeAST-6 (circles) superimposed on average annual mean SeaWiFS chlorophyll *a* concentration (mg. m^-3^). Filled symbols indicate locations where protein samples were collected.

Samples were collected during three cruises in the tropical and subtropical Atlantic (Figure 1). D326, a SOLAS (Surface Ocean Low Atmosphere Study) research cruise South of the Canary Islands, occurred during January-February 2008. FeAST-6, a cruise south of Bermuda, took place during summer (July) 2008, and AMT19 (Atlantic Meridional Transect), took place during Autumn (Oct.-Dec.) 2009. During these cruises, *Trichodesmium* spp. colonies were collected using a drift-plankton net (50 µm on AMT-19, 60 µm on D326 and FeAST-6) deployed at 5 m depth. Samples were collected at midday (AMT-19 and D326) and pre-dawn (FeAST-6). For protein analysis, colonies were picked individually, vacuum filtered with a hand pump onto glass fiber filters (0.3 µm nominal pore size, Advantec, Southampton, United Kingdom), snap-frozen in liquid nitrogen and stored at -80°C. The time from collection to freezing was always less than 30 min to avoid significant changes in the protein profile of the samples. Protein samples were collected from 1, 5 and 10 locations as part of the D326, FeAST-6 and AMT19 cruises, respectively. During D326, samples were also collected from an additional 7 stations to measure colony chlorophyll, particulate organic carbon and N_2_ fixation rates.

### Analytical Methods: N_2_ Fixation

During D326, N_2_ fixation was measured on samples of 20 picked *Trichodesmium* colonies following [55], acknowledging the potential for measured rates to be lower bounds due to incomplete equilibrium of the injected ^15^N_2_ gas with the sample [39]. Samples were analyzed by isotope ratio mass spectrometry calibrated against known carbon and nitrogen standards to derive colony particulate carbon, nitrogen and N_2_ fixation rates and hence, assuming steady state, estimate growth rates.

### Chlorophyll- *a* Measurement

For culture studies, 2 ml of cell cultures were filtered onto Whatman GF/F filters and snap frozen in liquid nitrogen pending analysis. For *in situ* samples, 5–20 colonies were filtered onto Whatman GF/F filters and immediately extracted. Filters were incubated into 4–6 ml 90% acetone overnight at 4°C in the dark and fluorescence was then measured according to [56].

### PSII Variable Chlorophyll Fluorescence

The photosynthetic physiology of natural *Trichodesmium* colonies was measured using active chlorophyll fluorescence on a Satlantic Fluorescence Induction and Relaxation (FIRe) [57], to determine the apparent photochemical quantum efficiency (F_v_/F_m_). For culture studies, PSII fluorescence of *Trichodesmium* IMS101 was measured once a day, 2 h after the onset of photoperiod, using a Fasttracka^TM^ Mk II Fast Repetition Rate fluorometer (FRRf) integrated with a FastAct^TM^ Laboratory system (Chelsea Technologies Group LTD, West Molesey, Surrey, United Kingdom). F_v_/F_m_ was taken as an estimate of the apparent PSII photochemical quantum efficiency [58]. For cultures, all parameters were measured directly after sampling, without dark adaptation under increasing background irradiance levels from 5 to 30 µmol photons m^-2^ s^-1^, which initially caused an increase in quantum yield. Data presented thus correspond to a background irradiance of 21 µmol photons m^-2^ s^-1^ (PAR).

### Total Protein Extraction and Quantification

Filters with 100–150 colonies (field samples) or 40–45 ml of culture (∼5000 trichomes) (culture samples) were resuspended in 1 ml denaturing extraction buffer containing 140 mM Tris base, 105 mM Tris-HCl, 0.5 mM EDTA, 2% lithium dodecyl sulfate, 10% glycerol, and 0.1 mg ml^-1^ PefaBloc SC protease inhibitor (Sigma-Aldrich, Pool, United Kingdom). The proteins were extracted according to the protocol described by [48]. Samples were concentrated on Centricon columns (3 kDa, Millipore, Watford, United Kingdom) from 1 ml to 250 µl. Total protein concentrations were measured using a modified Lowry assay (Bio-Rad, Hemel Hempstead, United Kingdom) with bovine γ-globulin as a comparative protein standard.

### Target Protein Quantification

**Figure 2:**
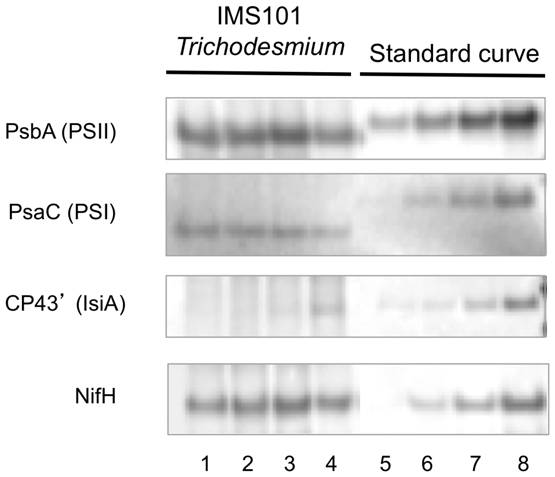
Immunoblots of targeted proteins in *Trichodesmium* IMS101 cultures. The first four lanes [1]–[4] correspond to individual cultures grown in media with initially increasing amounts of added Fe (200, 120, 40 and 20 nM respectively). Lanes for each antibody target were loaded with equal amounts of total protein. Immunodetection of synthesized peptides standard curve is presented in the last four lanes [5]–[8].

Key protein quantification was performed using standards (AgriSera, Vännäs, Sweden) and followed the procedure described by [48] and [59]. Primary antibodies (AgriSera, Vännäs, Sweden) were used in 2% ECL advance blocking reagent in Tris-buffered saline plus Tween 20 for NifH (Fe protein of nitrogenase), PsbA (D1 protein of PSII), PsaC (core subunit of PSI) and CP43’ (IsiA: iron-stress-induced protein A) for 1 h incubation. Blots were incubated for 1 h with horseradish peroxidase-conjugated chicken anti-rabbit secondary antibody (Abcam, Cambridge, United Kingdom) for PsbA, PsaC and CP43’ primary antibodies and with horseradish peroxidase-conjugated rabbit anti-chicken secondary antibody (Abcam, Cambridge, United Kingdom) for NifH. Blots were developed using ECL Advance detection reagent (Amersham Biosciences, GE Healthcare, Little Chalfont, United Kingdom) and a CCD imager (VersaDoc^TM^, Bio-Rad Laboratories Ltd, Hemel Hempstead, United Kingdom). For estimating the amounts of protein in experimental samples, protein levels on Millipore immunoblots were quantified using QuantityOne^TM^ and Image Lab^TM^ software and calculated from standard curves (for each blot, after [48]). Example protein detection images are shown in Figure 2.

### Redox kinetics of P700 Measured on Natural *Trichodesmium* Populations

**Figure 3:**
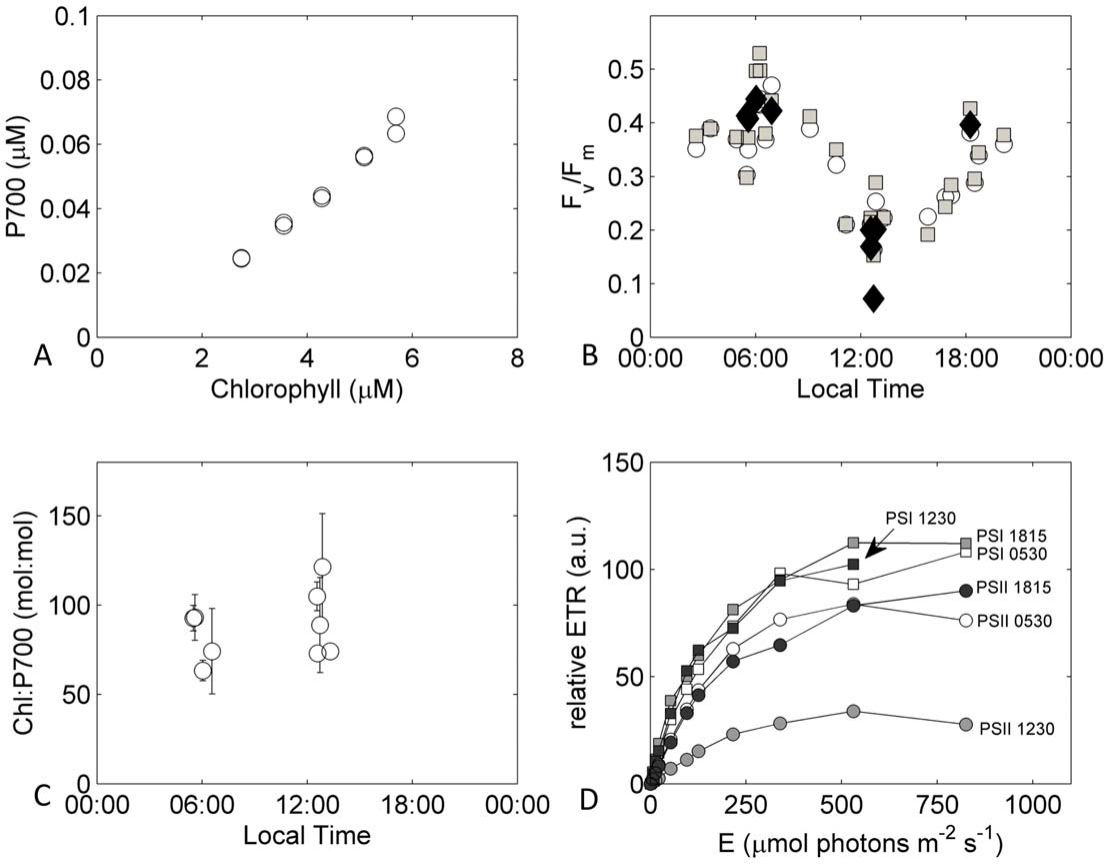
Photophysiological analysis of *Trichodesmium* colonies samples as part of D326. (A) correlation between chlorophyll and P700 concentrations for a single concentrated sample measured over a range of different dilutions allowing calculation of Chl:P700 (B) diel variability of F_v_/F_m_ measured on *Trichodesmium* colonies using Dual-PAM (filled diamonds) and both single (open circles) and multiple (closed squares) turnover measurements on a FIRe fluorometer, (C) variability in Chl:P700 measured directly on concentrated *Trichodesmium* colonies, and (D) relative electron transfer rate (ETR) through PSI and PSII complexes in colonies of *Trichodesmium* collected throughout a diel cycle calculated as the product of the respective photosystem quantum yield and irradiance.

During the D326 cruise, redox kinetics of the P700 reaction center chlorophyll of PSI were also measured on natural populations of *Trichodesmium* collected during net tows. To obtain sufficient biomass for measurements of absorption changes at 830 nm relative to 870 nm, samples were further concentrated by gentle filtration onto 10 µm polycarbonate filters followed by careful resuspension in the filtrate. Absorption changes at 830 nm (ΔA830) were measured alongside variable chlorophyll fluorescence in a Walz Dual-PAM 100™ (Heinz Walz GmbH, Germany). Comparison of F_v_/F_m_ values measured on concentrates and hand-picked colonies confirmed that the concentration step had no observable adverse effect on photochemical processes. A range of dilutions of concentrates were prepared for parallel measurement of ΔA830 and chlorophyll concentration to enable absolute quantification of the P700:Chl ratio for these natural populations (Figure 3A).

## Results

### PSII Photophysiology in *Trichodesmium* IMS101 and Natural Populations

**Figure 4:**
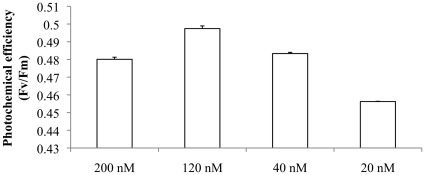
The influence of iron availability on PSII photochemical quantum yield (Fv/Fm) in *Trichodesmium* IMS101. Cultures were grown in media with initially increasing amounts of added Fe [200, 120, 40 and 20 nM]. Data obtained under background irradiance of 21 µmol photons m^-2^ s^-1^ (PAR). Significant differences between groups were tested by one-way ANOVA (p<0.01). Error bars indicate ± SD (n = 3).

After a 7- to 9-day incubation period, *Trichodesmium* cultures grown at the lowest levels of added Fe (20 nM Fe) exhibited decreased F_v_/F_m_ when compared with cultures grown at higher iron concentrations (Figure 4). Although we cannot quantify the relative iron availability within the cultures at the point of harvest, over the range of added Fe levels physiologies would have varied from iron-replete to iron-starved. As mentioned above, cultures with no added Fe did not grow. The maximal F_v_/F_m_ value was observed at 120 nM Fe. Data for cultures grown at the highest added Fe concentration (200 nM) indicated slightly suppressed F_v_/F_m_ compared to 120 nM.

Maximal daily values of F_v_/F_m_ measured on natural populations ranged from around 0.47 to 0.37 (data from cruise D326 and FeAST-6). As previously observed [34], F_v_/F_m_ measured in *Trichodesmium* spp. natural populations displayed a distinct diel cycle, with minimal values observed around 1 h after local noon (Figure 3B). In contrast, the quantum yield of PSI photochemistry displayed no clear diel cycle (Figure 3C,D). Consequently light response curves for PSII and PSI electron transfer rates (ETR) differed (Figure 3D), with relative PSI ETR measured at high saturating irradiance remaining constant throughout the photoperiod, while PSII electron transport reduced around midday (Figure 3D), coincident with likely maximal rates of N_2_ fixation [34].

### Abundance of Major Iron-binding Proteins in *Trichodesmium* IMS101 and Natural *Trichodesmium* spp. Populations

**Figure 5:**
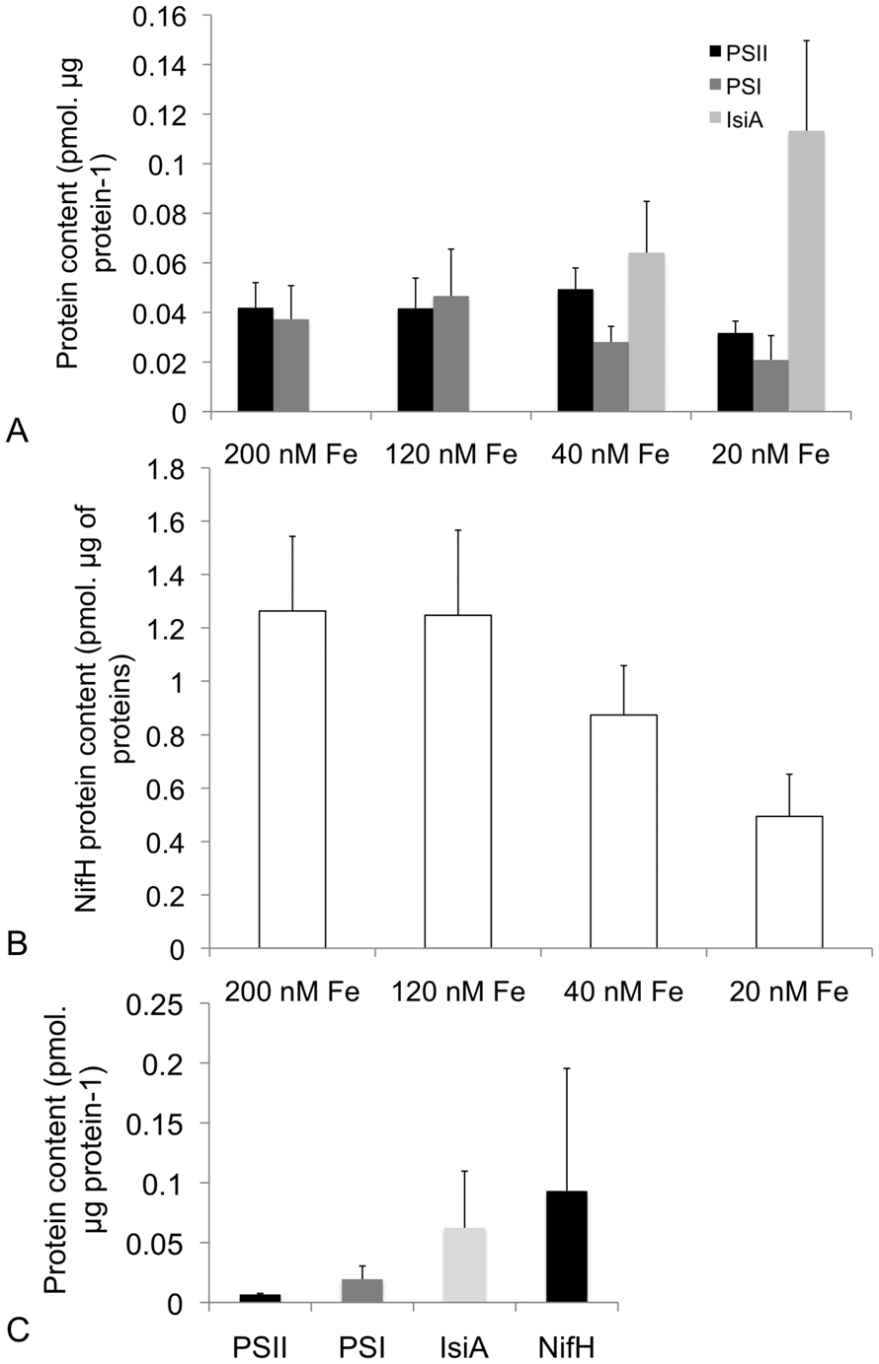
Changes in the absolute abundances of proteins of interest. (A) photosynthetic chlorophyll–binding proteins (PSI and PSI), iron-stress-induced protein IsiA, and **(**B) NifH protein in response to reduced iron availability in *Trichodesmium* IMS101 culture, and **(**C) iron-binding proteins (PSI, PSII and NifH) and iron-stress-induced protein IsiA in *Trichodesmium* spp. natural colonies. Significance between groups was tested by one-way ANOVA (p<0.01). Error bars indicate ± SD (n = 5 for (A) and (B) and n = 16 for (C).

Absolute protein concentrations obtained from quantitative Western-blot analysis were used to determine the photosynthetic/nitrogen-fixation protein profiles of *Trichodesmium* IMS101 harvested after 7 to 9 days growth. The level of iron starvation had little effect on the abundance of PsbA (D1 protein of PSII; Figure 5A); PsbA amounts decreased from 0.041 ± 0.001 pmol (µg total protein)^-1^ for the iron-replete culture to 0.031 ± 0.005 pmol (µg total protein)^-1^ under the most iron-starved condition. PsaC, a subunit of PSI, was more affected by Fe availability, decreasing from 0.037 ± 0.01 pmol (µg total protein)^-1^ under iron-replete conditions to 0.02 ± 0.01 pmol (µg total protein)^-1^ under iron-starvation (Figure 5A). The relative abundance of the two photosystems, PSI:PSII, estimated from the ratio of PsaC:PsbA, thus decreased with decreasing Fe availability across 4 culture conditions (one-way ANOVA p<0.1, Table 1). The Fe protein subunit of nitrogenase (NifH) was also influenced by Fe availability (Figure 5B). The NifH protein pool size was the largest for the cultures initiated with 200 nM of added Fe (1.26 ± 0.28 pmol (µg total protein)^-1^) and decreased significantly by more than 2-fold under iron starvation (0.49 ± 0.1 pmol (µg total protein)^-1^ at 20 nM Fe) (one-way ANOVA, p<0.01). The iron-stress-induced chlorophyll-binding protein IsiA was undetectable under iron-replete conditions. However, after 7–9 days we observed significant accumulation of IsiA for cultures initiated under the two lowest levels of added Fe (Figure 5A). IsiA protein abundance and the IsiA:PSI ratio thus both appeared to increase with increasing levels of iron stress (Figure 5A; Table 1).

**Table 1:** Selected subunit ratios for *Trichodesmium* IMS101 grown under different concentrations of added Fe.

	*Trichodesmium* IMS101 [culture]			
	[200 nM] Fe	[120 nM] Fe	[40 nM] Fe	[20 nM] Fe
PSI:PSII	0.9 ± 0.5	1.1 ±0.3	0.6 ± 0.2	0.6 ±0.3
IsiA:PSI	u.d.	u.d.	2.6 ± 1.6	6.4 ±3.3
IsiA:PSII	u.d.	u.d.	1.33 ± 0.25	3.9 ± 1.1

u.d. indicates that IsiA was undetectable at the two lowest concentrations of iron.

*Trichodesmium* colonies were collected using standardized protocols during three different cruises (Figure 1). From these samples, the absolute abundances of PSI, PSII, NifH and IsiA were measured (Figure 5C, Table 2). Considerable variability in estimated absolute abundances was observed for field samples (Table 2), with no statistically distinguishable spatial or temporal coherence within or between cruises. In the absence of significant trends, we thus consider the average protein profile from all the collected colonies of *Trichodesmium* in the subtropical North Atlantic Ocean across all locations (n = 16 independent samples) (Figure 5C). Consistent with protein profiles obtained from cultures, NifH was the most abundant of the quantified proteins (Figure 5C). The iron-stress-induced protein IsiA was always present and was more abundant than either of the photosynthetic reaction centers PSI or PSII (Figure 5C). Overall, the protein profile of natural communities was more similar to that obtained from iron-starved than iron-replete cultures, largely reflecting the presence of IsiA and the abundance of this protein relative to the photosystem subunits (Figure 5C). The quantity of the NifH protein in natural communities, ranged from 0.02–0.3 pmol (µg total protein)^-1^ (Figure 5C), similar to that measured on iron-starved cultures of *Trichodesmium* IMS101 (0.49 ± 0.1 pmol. µg total protein^-1^) and never approached the concentrations per unit protein observed in iron-replete cultures (Figure 5B).

**Table 2:** Selected subunit absolute quantification and ratios for *Trichodesmium* spp.

	*Trichodesmium* spp. [natural populations]			
Cruise	AMT-19	FeAST-6	D326	all
PSI (pmol (µg of protein)^-1^)	0.005 ± 0.005	0.004 ± 0.005	0.049 ± 0.02	0.019 ± 0.011
PSII (pmol (µg of protein)^-1^)	0.007 ± 0.005	0.008 ± 0.004	0.004 ± 0.003	0.007 ± 0.001
IsiA (pmol (µg of protein)^-1^)	0.034 ± 0.02	0.032 ± 0.04	0.12 ± 0.1	0.06 ± 0.05
NifH (pmol (µg of protein)^-1^)	N/A	0.132 ± 0.1	0.054 ± 0.002	0.09 ± 0.1
PSI:PSII	1.0 (0.3 – 1.7)	1.4 (0.6 – 2.2)	5.9 (4.6 – 7.3)	2.0 (0.3 – 7.3)
IsiA:PSI	4.1 (2.3 – 8.0)	7.5 (1.7 – 19.4)	1.97 (1.6 – 2.4)	5.0 (1.7 – 19.4)
IsiA:PSII	3.9 (2.2 – 6.7)	7.7 (0.7 – 23.6)	16.2 (11.0 – 22.5)	8.5 (0.7 – 23.6)

Colonies collected from 1, 5 and 10 locations as part of the D326, FeAST-6 and AMT19 cruises, respectively. For proteins absolute values, SD correspond to n = 16 samples. For ratios, average means are presented along with the corresponding range into brackets.

### Chlorophyll Budget in Culture and Natural Populations of *Trichodesmium* spp

Quantification of all the major chlorophyll-containing complexes enabled us to derive a cellular chlorophyll budget. The presence of phycobilisomes as the major light-harvesting complex in *Trichodesmium*, results in the majority of chlorophyll in cyanobacteria being bound to the reaction centers PSI and PSII or, under iron-stress, potentially to IsiA [52]. The number of chlorophyll molecules in each of the complexes has been determined through high-resolution structural analysis of the photosynthetic reaction centers [16], [60], [61] [Chl_PSII_ = 36; Chl_PSI_ = 100; Chl_IsiA_ = 12]. These values were thus used to derive ratios of total cellular chlorophyll to each complex, and hence also estimate the relative contribution (expressed as a %) of the total cellular chlorophyll bound within each of the complex pools [52]. For example, assuming 1 PsbA per PSII, and 1 PsaC per PSI, the ratio of total cellular chlorophyll to the cellular concentration of PSI (Chl:PSI) can be estimated from:
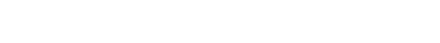
The % of total cellular chlorophyll bound to PSI can further be calculated as:
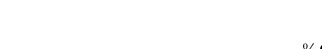
with similar equations derivable for PSII and IsiA:
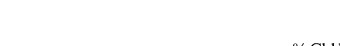


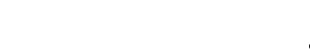



Thus, for example, estimated ratios of Chl:PSI ranged from 150 mol:mol for iron-replete cultures to 800 mol:mol for iron-starved cultures where chlorophyll bound to IsiA significantly contributed to the total cellular budget, while the calculated value for Chl:PSI was 180 mol:mol for average field populations. As an independent check, during the D326 cruise spectrophotometric measurements averaged 93 ± 30 Chl:P700 (Figure 3A and 3C), compared to 130 Chl:PsaC as derived from protein ratios.

**Figure 6:**
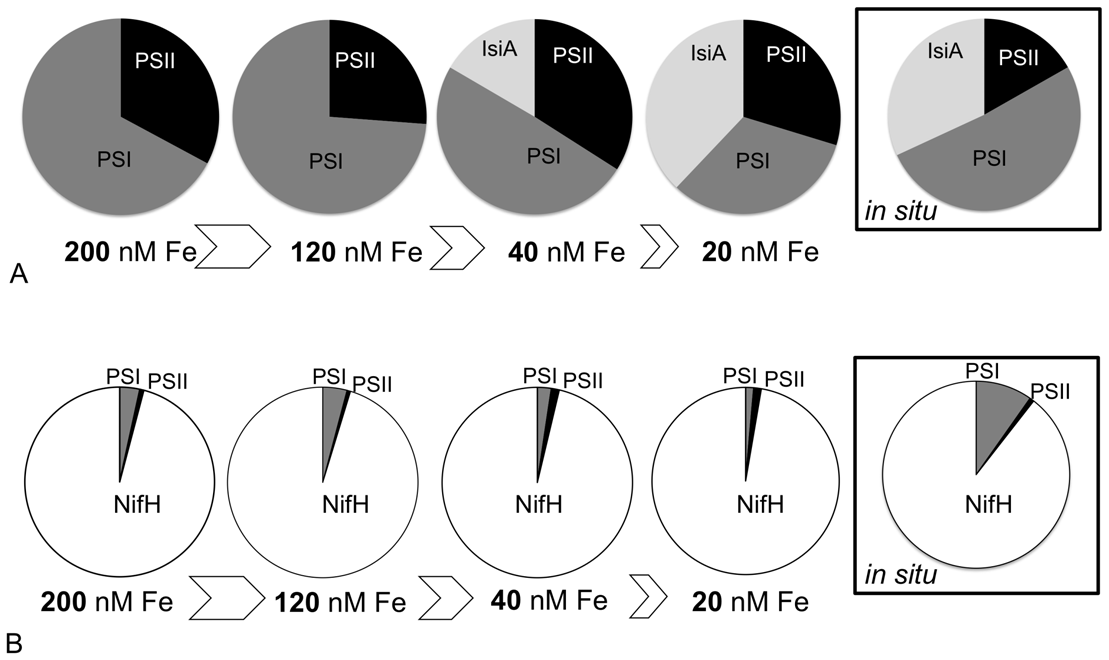
Chlorophyll and Fe Budget in culture and natural population of *Trichodesmium* spp. Chlorophyll budget (A) and Fe partitioning (B) in the evaluated major Fe-binding proteins of *Trichodesmium* IMS101 grown in culture media with initially different concentrations of added Fe and in *Trichodesmium* spp. natural populations (box). Pie charts represent the percentage of either chlorophyll or Fe in each of the protein complexes. Calculations in (B) assume 19 Fe atoms within the whole nitrogenase complex per NifH (see text).

From the above equations, chlorophyll budgets for *Trichodesmium* in culture and in the field were thus compared (Figure 6A). Chlorophyll partitioning between the main chlorophyll-binding complexes in *Trichodesmium* IMS101 (e.g. PSI, PSII and IsiA) was significantly modified by iron starvation, partly due to a reduced PSI:PSII ratio, but principally due to the shift from two (PSI and PSII) to three (PSI, PSII and IsiA) main chlorophyll complexes (Figure 6A). The percentage of chlorophyll associated with PSII complexes remained relatively unchanged (25-33%); consequently, we effectively observed a re-allocation of total cellular chlorophyll from PSI (decreasing from 75% to 34%) to the IsiA complex (increasing from 0% up to 38%). Interestingly, the average chlorophyll % associated with each of the complexes in natural populations of *Trichodesmium* collected from the North Atlantic approximated that within iron-starved cultures, with 17%, 51% and 32% chlorophyll allocated to PSII, PSI and IsiA, respectively.

### Partitioning of Fe Atoms between the Major Iron-binding Proteins in Culture and Natural Colonies of *Trichodesmium* spp

In a similar manner to the derived cellular chlorophyll budgets, the proportions of Fe in different cellular metabolic pools were calculated based on estimates of Fe atoms associated with each complex from structural studies. PSII and PSI have 3 and 12 Fe atoms per complex respectively [48], [19]. Further, to more fully account for additional Fe within the photosynthetic electron transport chain, we assume a ratio of 1∶1 for the Cytochrome b_6_f:PSI ratio, with 6 Fe atoms per Cytochrome b_6_f (Cytb_6_f) complex [18]. Consequently we assume a total of 18 Fe atoms per Cytb_6_f+PSI. The total Fe content of the nitrogenase complex depends on the ratio of Fe to MoFe protein (coded for by *nifH* and *nifDK* respectively) which has not to our knowledge been measured in *Trichodesmium*. Maximal specific activity for nitrogenase appears to occur at a ratio near 5 Fe:MoFe [62], [63], with structural analysis of the protein [20] and *nifH/nifDK* transcript products [64] indicating a range of ratios from 2–5. This corresponds to a possible Fe content of 38–50 mol Fe (mol nitrogenase complex)^-1^, i.e. a factor of 2 variability in Fe content. As we measure the NifH complex, we thus estimate a potential range of total Fe in nitrogenase: NifH of 10–19 mol Fe (mol NifH)^-1^. We consequently perform calculations of Fe allocation across the different metabolic complexes for the upper and lower end of the ranges indicated above. For example, taking 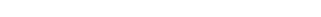. we can derive the following:
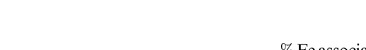


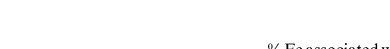


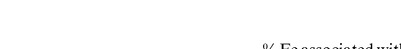



Clearly there are likely to be other unaccounted for cellular Fe requirements, such as Fe superoxide-dismutase [18]. Our estimates thus represent lower bounds for the total cellular Fe budget for *Trichodesmium*. In a manner similar to [28], we thus consider such estimates to represent the Fe metalloenzyme or metabolic inventory associated with the cellular processes of N_2_ fixation and photosynthesis, although, as argued below, this potentially accounts for the majority of cellular Fe.

The percentage of Fe in the major iron-binding complexes was compared between culture and natural populations of *Trichodesmium* spp. (Figure 6B). Based upon the relative abundance of PsaC, PsbA and NifH, the major metabolic iron inventory (83–95% of Fe) under all culture conditions was estimated to be associated with the nitrogenase enzyme complex (Figure 6B). An inverse relationship was also observed between iron stress and iron allocation to PSI. The same analysis performed on average values derived from natural populations of *Trichodesmium* spp. indicated a similar partitioning of Fe between the resolved metabolic activities, with 9–15% Fe bound to the PSI complex versus 83–90% for the nitrogenase complex.

### Comparison of Theoretical and Prior *in vitro/in vivo* Data

Combining our derived Fe and Chl budgets, we further calculate the cellular metabolic Fe:Chl ratio. The combined influence of IsiA accumulation and a reduction in nitrogenase (Figure 5) resulted in estimated cellular metabolic Fe:Chl reducing from 1.7–3 mol:mol under iron-replete conditions to 0.2–0.34 mol:mol under iron-starved conditions, with all quoted ranges again indicating the sensitivity of calculations to the assumed Fe:MoFe protein (*nifH/nifDK* transcript product) ratio. For comparison, the average value estimated from our measurements of natural populations was 0.3–0.5 mol:mol. Although significant variability in cellular Chl:C needs to be acknowledged, assuming Chl:C ratios of ∼200 µmol:mol [25], [18], [65], we estimate that metabolic Fe:C ratios could have increased in *Trichodesmium* ISM101, from around 40–68 µmol:mol under iron-stressed conditions to 340–600 µmol:mol under iron-replete conditions (Table 3). These values were very comparable with previous direct measurements of total cellular Fe:C (Table 3) on cultures [41], [44] and to a previous metabolic estimate of 236 µmol Fe bound to nitrogenase (mol C)^-1^ [66], consistent with ∼80% of the metabolic cellular Fe pool we quantify being associated with N_2_ fixation (Figure 6B).

**Table 3:** Some characteristics of culture and natural populations of *Trichodesmium* spp.

	Culture				Natural populations from theNorth Atlantic	
	Iron replete		Iron stressed			
	Total	Metabolic	Total	Metabolic	Total	Metabolic
Chl:Col (ng col^-1^)					*6–60* a; 8–60^b^	
C:Col (µg col^-1^)					*3–6* a *;*2–15^b^;1–4^c^	
Fe:Col (pmol col^-1^)					4–14^c^	*2–35* a
Chl:C (µmol mol^-1^)	300–350^d^; 325^e^		175–200^d^; 121^e^		*94–121* a; 20–247^b^	
Fe:C (µmol mol^-1^)	200–500^d^; 168^e^	*340–600* a; 236^f^	20–40^d^; 13^e^	*40–68* a	20–175^d^; 20–80^c^	*15–75* a

a This study, see text.

b Carpenter et al. 2004.

c Sanudo-Wilhemy et al. 2001.

d Kustka et al. 2003b.

e Berman-Frank et al. 2001a.

f Whittaker et al. 2010, note estimate is for iron bound to nitrogenase only.

Colony chlorophyll contents measured at multiple stations on D326 ranged from 6–60 ng Chl colony^-1^ (16 ± 14, mean ± 1 s.d., n = 33 samples, 7 stations, Table 3). Particulate organic carbon contents measured at a more limited number of stations ranged from 3–6 µg C colony^-1^ (4.8 ± 1.4, n = 4), which, combined with an average of 37 ± 11 ng Chl colony^-1^ at these same locations, resulted in an estimated Chl:C ratio of 107 ± 20 µmol:mol. These values are consistent with previous observations of Chl:C ratios for natural *Trichodesmium* colonies [67] and comparable, although slightly lower than, values for diazotrophically growing cultures [44], the lower values *in situ* potentially reflecting a contribution from other microbes within the colony matrix [68]–[70].

Combining our estimates for metabolic Fe:Chl ratios of 0.3–0.5 mol:mol with measured colony Chl contents, we estimate a conservative range for the metabolic Fe content of *Trichodesmium* of 2–35 pmol Fe colony^-1^ (average 7 pmol Fe:colony), compared with direct estimates of 4–12 pmol Fe colony^-1^ [11]. Measured Chl:C ratios further suggest 15–75 µmol metabolic Fe (mol C)^-1^ for natural *Trichodesmium* populations, which is again comparable with previous direct elemental ratios measured on both natural populations and iron limited cultures [44]. Overall estimates of the metabolic iron demand were thus consistent with the majority of the cellular pool being associated with the metalloenzymes of the photosynthetic and nitrogen fixation apparatus (Table 3).

Total *Trichodesmium* chlorophyll standing stocks ranged from <0.0001 µg Chl l^-1^ to maximal values of 0.07 µg Chl l^-1^ in the subtropical North Atlantic during February-March 2008, with direct estimates of chlorophyll in the >10 µm fraction measured on a 10 L sample being of a similar magnitude. Bulk community *in situ* chlorophyll concentrations (i.e. >0.2 µm measured on 200 ml of a whole water sample) thus indicated that *Trichodesmium* accounted for up to 20% of the total chlorophyll standing stock at some stations. Over the observed ∼50m mixed layers, maximal *Trichodesmium* concentrations of 0.07 µg Chl l^-1^ during D326 thus represented standing stocks of 3.5 mg *Trichodesmium* Chl m^-2^. When combined with our estimates of cellular metabolic Fe requirements, this was thus the equivalent of a metabolic Fe standing stock of 1–2 µmol m^-2^ (Table 4).

**Table 4:** Comparison of theoretical and *in situ* data related to culture and natural population of *Trichodesmium* spp. from North and South Atlantic Ocean.

	Theoretical	North Atlantic	South Atlantic
µ (d^-1^)	At 0.1	0.02–0.05^b^ *	
Fe:C (µmol:mol)	28–40^a^	15–75^b^	
*Trichodesmium* standing stock (ug Chl m^-2^)		3500^b^	<3.5^c^
Metabolic Fe standing stock (nmol m^-2^)		1000–2000^b^	1–2^b^
Fe requirement (nmol m^-2^ d^-1^)		50–100^b^ *	0.05–0.1^b^
Fe deposition (nmol m^-2^ d^-1^)		100–1000^d^150^e^	<50^d^1–20^e^

* Values likely represent a lower bound due to the potential for incomplete equilibration of ^15^N_2_ over the duration of the incubation (Mohr et al. 2010).

a Kustka et al. 2003a.

b This study.

c Moore et al. 2009.

d Baker et al. 2003.

e Mahowold et al. 2009.

## Discussion

In cultures of *Trichodesmium* IMS101, the onset of iron starvation was indicated by a decline in photochemical efficiency (F_v_/F_m_), as previously shown [25], [41]. Although comparison of absolute values of F_v_/F_m_ will be complicated by a range of other growth factors, *in situ* values measured on natural populations of *Trichodesmium* were consistent with those previously reported in the literature [34] and most comparable to iron-starved cultures.

The photosynthetic/nitrogen-fixation protein profiles of *Trichodesmium* either in culture or in the field show a high ratio of NifH to PsbA and PsaC proteins (Figure 5B and 5C), potentially reflecting a lower enzymatic rate for nitrogenase compared to photosynthetic proteins [48]. Our results reveal a small decrease in abundance of photosystem I and II complexes, but a much more significant decline in nitrogenase in iron-starved cultures. These results corroborate the trends observed at a gene-expression level by [19] who documented an early decline in *nifH* transcripts during the development of iron stress followed by a later decline in transcripts encoding the photosynthetic apparatus. Consequently it appears that the metabolic process of N_2_ fixation may be more sensitive to iron availability than photosynthesis [19]. In addition, PSII appears to be less sensitive to iron limitation than PSI at both gene and protein levels [19], [48]. Our results indicate that the nitrogenase enzyme is the major metabolic sink for iron in *Trichodesmium* cells. Transfer of iron from the energetically and iron-expensive processes of nitrogen fixation to maintain energy production via the photosynthetic apparatus [19], [47] could thus be speculated to act as a mechanism for surviving in oligotrophic subtropical and tropical areas characterized by fluctuations of iron supply [71].

Nitrogen fixation has been studied extensively in the tropical and subtropical North Atlantic [4], [5], [14], [67]. Due to the proximity of African deserts, most significantly the Sahara, the area is subject to high rates of dust deposition [71]–[75], which likely result in the observed high surface iron concentrations [14]. Indeed, the low latitude North Atlantic has the highest known Fe concentrations of any of the global subtropical basins [15]. Despite this relatively high iron availability, we still found potential protein level evidence of reduced iron requirements within natural *Trichodesmium* populations based on comparisons with the cultured strain IMS101.

Significant caveats clearly need to be acknowledged when comparing culture results from a starvation experiment, run on a specific strain, under a limited set of other culture conditions, to field populations experiencing variable additional environmental forcings. Thus, although N_2_-fixing enzyme abundances were in similar range in both *Trichodesmium* natural populations and in iron-starved cultures, phosphate is also severely depleted in the subtropical North Atlantic gyre, likely as a result of enhanced N_2_ fixation due to dust deposition [14], [76]. Consequently, natural *Trichodesmium* colonies from the region frequently display evidence of phosphate stress [13], [50], [77]–[78], which may thus contribute to any reduced capacity for N_2_ fixation [11].

The IsiA protein was also present in all the natural populations sampled (Figure 5C) and is expressed [19] or accumulates (Figure 5A) under development of iron stress in *Trichodesmium* cultures. Although IsiA may be expressed under other growth conditions, including high light [79], acclimation to iron stress appears to be the primary functional role for this chlorophyll binding complex [47], [80]–[83], which can act as a light-harvesting antenna for PSI [52], [82].

In the present study, the IsiA protein constitutes a significant portion of the chlorophyll-binding protein in iron-starved cultures of *Trichodesmium* ISM101 and in natural populations (Figure 6A), with up to 4 and 6 times more IsiA than PSI and PSII proteins in starved cultures and natural populations, respectively.

The observed maintenance of PSI electron transport throughout the photoperiod in natural communities, in contrast to the down-regulation of electron transport through oxygen-evolving PSII (Figure 3D), supports the suggestion that PSI electron transport may act to consume cellular O_2_ in order to protect nitrogenase from inactivation in *Trichodesmium* [33]. A subsequent requirement for maintenance of cellular PSI concentrations may further enhance the iron requirements of this organism compared with other diazotrophs [41]. For example, *Crocosphera watsonii* separates N_2_ fixation and oxygenic photosynthesis over the diel period [34] and hence can effectively share cellular Fe between these molecular processes [28]. Maintenance of PSI electron transport may also provide a rationale for increasing the light-harvesting cross-section of PSI in *Trichodesmium* under conditions of reduced iron availability through the expression of IsiA and the synthesis of IsiA-PSI supercomplexes [52], [84].

Similar to previous calculations [25], [66] estimation of iron within the molecular mechanisms of N_2_ fixation and photosynthesis provides a means of extrapolating to the natural environment. Our estimate of 80–90% of the cellular Fe pool being associated with nitrogenase is consistent with theoretical calculations suggesting that 35–78% of the cellular Fe pool would be associated with N_2_ fixation under optimal catalytic conditions [18]. Kustka et al. [18] estimated that cellular Fe:C ratios of 28–40 µmol:mol would be required to maintain a moderately iron-limited growth rate of around 0.1 d^-1^, consistent with both laboratory studies [41] and our observations of natural populations (Table 4). Combining observed nitrogen specific N_2_ fixation rates with metabolic iron standing stocks, natural populations of *Trichodesmium* in the region sampled during D326 would hence require 50–100 nmol Fe m^-2^ d^-1^ (Table 4). Cautious comparison with culture data (Table 3) further indicates that iron requirements could potentially be an order of magnitude higher under fully iron replete conditions. Observed ten-fold higher standing stocks of *Trichodesmium* in other regions of the North Atlantic [67], would also require correspondingly higher Fe turnover.

Although iron is known to be readily recycled in the upper ocean, knowledge of the differential cycling of nutrients (i.e. N or Fe) in oligotrophic systems is incomplete [85]. Given that the N_2_ fixed by diazotrophs represents a source of ‘new’ nitrogen (*sensu* [86]) to first order it is reasonable to postulate that new iron inputs [85] may be required to balance much of the daily diazotrophic requirement. Given our estimated metabolic Fe demands, atmospheric dust related inputs of dissolved iron to the subtropical North Atlantic would thus be sufficient to satisfy the requirements of the large standing stocks of *Trichodesmium* observed in this region (Table 4). However, inputs would potentially be insufficient to satisfy the requirements of a metaloenzyme composition equivalent to that of an iron-replete culture.

Simple ecological theory predicts that the rate of supply of a limiting resource will control the organism standing stock, while the ecophysiological characteristics of these organisms will determine the ambient concentration of the resource [29], [87], [88]. We thus suggest that the response of *Trichodesmium* to atmospheric dust inputs to the North Atlantic [14] may lead to population growth and subsequent iron uptake reducing bioavailable levels to the point necessitating some reduction of cellular requirements. Such biological control of iron availability by the diazotrophic population would, however, be unlikely to result in severe stress and heavily reduced growth, a scenario which is consistent with the lack of expression of biomarkers of high iron stress in the region [46], [50]. Other factors will clearly complicate this simple scenario, including non-biological influences on surface iron bioavailability [84] and, particularly in the North Atlantic, depletion of phosphorous [11], [12], [50], [76]. However, consistent with previous experimental work [12], [50], it appears that a limited degree of diazotrophic iron stress may potentially develop even in some regions of high input.

In contrast to the North Atlantic, other subtropical oceanic regions receive much lower iron inputs, which may thus represent a severe constraint on the accumulation of high standing stocks of *Trichodesmium* [15], [25], [71]. For example, *Trichodesmium* biomass [14] and hence estimated iron requirements are 3 orders of magnitude lower in the subtropical South Atlantic (Table 4). Our molecular characterization of the metabolic pools within natural populations of *Trichodesmium* hence provides further evidence of an important role for iron in dictating the large scale biogeography of this important diazotrophic taxon [15], [25], [27], [29].

### Conclusion

Relative to other diazotrophs [28], [41], enhanced iron demand resulting from coordination of photosynthesis and nitrogen fixation may contribute to *Trichodesmium* being particularly sensitive to iron availability. In turn, high iron requirements have likely led to the evolution of both mechanisms for acclimating to reduced availability [19] and novel acquisition strategies [89]. Using a combination of laboratory and field experiments we suggest that natural *Trichodesmium* populations from the subtropical North Atlantic display molecular characteristics representative of a reduction in metabolic Fe-metaloenzyme requirements relative to iron-replete cultures. It thus appears that iron may influence *Trichodesmium* ecophysiology even in some regions receiving relatively high dust inputs. The current study adds further molecular level understanding to a growing body of evidence supporting the role of iron as a control on oceanic N_2_ fixation.
